# The use of a dietary quality score as a predictor of childhood overweight and obesity

**DOI:** 10.1186/s12889-015-1907-y

**Published:** 2015-06-24

**Authors:** Catherine P. Perry, Eimear Keane, Richard Layte, Anthony P. Fitzgerald, Ivan J. Perry, Janas M. Harrington

**Affiliations:** Department of Epidemiology and Public Health, University College Cork, 4th Floor Western Gateway Building, Western Rd, Cork, Ireland; School of Mathematical sciences 1st floor Western Gateway Building, Western Rd, Cork, Ireland; Economic and Social Research Institute (ESRI), Sir John Rogerson’s Quay, Dublin 2, Ireland; Department of Sociology, Trinity College Dublin, 3 College Green Dublin 2, Dublin 2, Ireland

**Keywords:** Diet score, Dietary quality score, Nutrition, Child, Obesity, Overweight, A-prori, Diet quality, Ireland

## Abstract

**Background:**

The use of dietary quality scores/indices to describe diet quality in children has increased in the past decade. However, to date, few studies have focused on the use of these scores on disease outcomes such as childhood obesity and most are developed from detailed dietary assessments. Therefore, the aims of this study were: firstly to construct a diet quality score (DQS) from a brief dietary assessment tool; secondly to examine the association between diet quality and childhood overweight or obesity; thirdly we also aim to examine the associations between individual DQS components and childhood overweight or obesity.

**Methods:**

A secondary analysis of cross sectional data of a sample of 8,568 9-year-old children and their families as part of the Growing Up in Ireland (GUI) study. Subjects were drawn from a probability proportionate to size sampling of primary schools throughout Ireland over the school year 2007–2008. Height and weight were measured by trained researchers using standardised methods and BMI was classified using the International Obesity Taskforce cut-points. The DQS (un-weighted) was developed using a 20-item, parent reported, food frequency questionnaire of foods consumed over the past 24 h. Adjusted odds ratios for overweight and obesity were examined by DQS quintile, using the first quintile (highest diet quality) as the reference category.

**Results:**

The prevalence of normal weight, overweight and obese was 75, 19 and 6 % respectively. DQS ranged from -5 to 25, higher scores indicated higher diet quality in the continuous score. In analyses adjusted for gender, parent’s education, physical activity and T.V. viewing, child obesity but not overweight was significantly associated with poor diet quality: OR of 1.56 (95 % CI 1.02 2.38) in the 5th compared to the 1st DQS quintile. Findings from individual food items were inconsistent.

**Conclusions:**

The findings suggest that diet quality may be an important factor in childhood obesity. A simple DQS developed from a short dietary assessment tool is significantly associated with childhood obesity.

**Electronic supplementary material:**

The online version of this article (doi:10.1186/s12889-015-1907-y) contains supplementary material, which is available to authorized users.

## Introduction/background

The prevalence of childhood overweight and obesity is a public health concern globally. More recent data state the prevalence of overweight or obesity is approximately one quarter of children worldwide [1, 2]. Ireland is no exception to this issue, a study tracking secular trends in the height and weight of Irish children from 1948–2002, found that the weight of Irish children had increased disproportionally to their height [3]. Obesity affects the physical and psychosocial wellbeing of children and is a significant risk factor for obesity in adulthood [4-8]. It also creates a significant social and financial burden for wider society [9].

Obesity is a complex issue arising from a myriad of individual and environmental factors. The imbalance between energy intake and energy expenditure is a central causal factor for obesity [10, 11] and therefore what we eat is important. Currently, there is no consensus on the best approach to measure the association between diet and childhood obesity. To describe this diet-disease association diet quality has gained increasing attention in the literature as a predictor of childhood obesity. However there is no agreement regarding the definition of diet quality [12]. Much of the adult literature uses dietary quality scores (DQS) or indices as a measure of diet quality [12]. DQS are also emerging in the paediatric literature [13] and more recently their association with health related outcomes in children has been reviewed [14].

Investigating dietary quality using DQS better reflects the real-life scenario that foods are not consumed in isolation [15, 16]. There are two forms of dietary pattern analysis: an a priori approach DQS, based on current recommendations or a known hypothesis of a healthy diet; and *an a posteriori* approach based on data driven statistical analysis [17, 15]. There is a need to measure the association between whole diet and childhood overweight and obesity. Adherence to dietary recommendations may be useful to provide an insight into dietary patterns in children. The a-priori approach is reproducible across populations and can be used to compare diets across different countries [18]. Studying children’s dietary patterns could have an important public health impact since results may be easier to translated into public health messages than research on specific foods or nutrients [19, 20].

Recent studies examining associations between diet quality and health outcomes including obesity in children are predominantly based on detailed dietary assessments [13] which have high respondent and researcher burden. There is a need to examine the potential value of simple dietary assessment tools from which simple DQS’ can then be developed to measure adherence to dietary recommendations. A systematic literature review stated that brief tools to assess diet quality should include the five major food groups [21]. These simple tools may be more suitable for use in large-scale epidemiological studies that collect data on all aspects of health and development. Therefore, the aims of this research are firstly to construct a diet quality score from a brief dietary assessment. Secondly, to examine the association between diet quality and childhood overweight or obesity. Thirdly this study also examines the associations between individual DQS components and childhood overweight or obesity.

## Methods

### Participants

Participants were recruited for a nationally representative longitudinal study, the Growing Up in Ireland (GUI) study. For the purpose of this research a secondary analysis of the first wave of the GUI study, 9-year-old cohort, completed over the 2007–2008 school year, was conducted. The objective of the GUI study was to chart the development of children over time, to examine the progress and wellbeing of children at critical periods from birth to adulthood and investigate what determines healthy or unhealthy child development. Details of the methodology have been published elsewhere [22, 23]. Briefly, a two-stage clustered sampling strategy was employed. A random sample of 1,105 primary schools was drawn on a probability proportionate to size sampling method (list obtained from the Department of Education and Skills website). Subject to the school’s participation, age-eligible children and their families were then invited to participate in the study [22, 23]. Parental consent and child assent forms were gathered and only those who returned forms were eligible to participate. There were 8,568 9-year-old children and their families recruited into the first wave of the study. At the school level, a response rate of 82 % was achieved, while at the household level (i.e. eligible child selected within the school) 57 % of children and their parents participated in the study. The data was reweighted to ensure representativeness of all 9-year-old children in Ireland.

### Procedures

Trained researchers went to the study child’s house to complete questionnaires with the study child and their parents/guardians. The person nominated as the primary care giver who spent the most time with the study child and was the biological mother in 98 % of cases. The primary caregiver is referred to as the parent throughout this manuscript.

### Anthropometric measures

Anthropometric measurements were obtained during the household interview using validated, standardised methods conducted by trained researchers [22]. Weight measurements of parents and children were recorded to the nearest 0.5 kg using a SECA 761 medically approved flat mechanical scales. Height was recorded to the nearest millimetre using a Leicester portable height stick. Valid height and weight measurements were obtained for 94.5 % of children and 91 % of parents. Reasons for exclusion of height and weight were; refusals, cases where it was not physically practical to take a measurement (e.g. person on crutches or in a wheelchair). Children were classified into Body Mass Index (BMI) categories using gender and the 9.5 year age specific, International Obesity Taskforce (IOTF) cut points [24], this was used as the outcome variable. Measured parent BMI was classified according to the World Health Organization (WHO) classifications as normal weight (<25 kg/m^2^), overweight (≥25 and <30 kg/m^2^) or obese (≥30 kg/m^2^) [25].

### Dietary assessment

For the purpose of the GUI study a 20 item, parent reported, Food Frequency Questionnaire (FFQ) of foods consumed over the past 24 h [23, 26], was used to estimate children’s diets. This FFQ will be referred to as a short FFQ throughout the rest of this manuscript. The Amherst Health and Activity (AHA) study questionnaire contributed to 7/20 of the questions and this questionnaire as a whole was evaluated elsewhere [27, 28]. Five questions were obtained from the Growing up in Australia Study [29]. The GUI study added 8 questions by consultation with the expert health panel set up by the GUI study team. The short FFQ was pilot-tested with parents [30]. Twenty food items were listed and the parent responded whether the study child did not eat/drink the items at all, ate food/drink items once, more than once or don’t know, over the previous 24-h, the dietary assessment tool can be found in (Additional file 1 (a)). The child’s diet was also assessed in the child questionnaire by a 10 item FFQ in which the child responded if they did not eat a food, ate one serving or ate more than one serving yesterday (Additional file 1 (b)). For this study, the parent reported dietary assessment was used in the main analyses and the child reported score was analysed and is displayed in the additional files. Analysis of the statistical reliability of the parent reported dietary index shows that it achieves an alpha coefficient of 0.47, which is comparatively low [26]. However, the GUI researchers did not include all the items to produce this alpha coefficient.

A form of data triangulation the GUI study used to check for consistencies in the dietary assessment was giving the parents and the children similar questions. The GUI researchers created indices of diet quality from both the parent and child reported dietary assessments. They summed the scores and got the mean value. Analysis of the indices found that the child index was weakly correlated with the parental index and displayed little structured variability [26].

### Dietary quality score

An un-weighted DQS was constructed from the short FFQ. Foods were deemed healthy or unhealthy based on current Irish guidelines and guided by Food Safety Authority of Ireland recommendations [31]. ‘Healthy foods’ were given a value of 0 if not eaten at all, 1 if eaten once and 2 if eaten more than once. ‘Unhealthy foods’ were negatively scored and assigned values of −2 if eaten more than once, −1 if eaten once and 0 if not eaten at all. For scoring purposes, ‘don’t know’ answers from the FFQ were recoded to missing. Missing data for the score were scaled to the mean answers when there was less than or equal to two answers ‘missing’ in the healthy item score and less than or equal to two missing answers in the unhealthy item score, this accounted for a very small percentage of people (*n* = 68/8568 = 0.8 %). The component scores across the 20 food items were then summed to get an overall DQS, which, could theoretically range from −12 to 28, with higher scores indicating a better diet. The score was also divided into quintiles, quintile 5 indicates the poorest dietary quality and quintile 1 indicates the highest in the categorical format. Secondary to the parent reported analyses; the same method was employed to create a DQS for the child’s self-reported diet and this was used to investigate if marked differences between parent and child reported DQS were evident.

### Covariates

Child’s gender (boy/girl), physical activity (PA) television (TV) viewing, and parent’s highest level of education were obtained from the parent questionnaire. Child’s PA was measured from the question “number of times in the last 14 days study child did hard exercise” responses were coded as; none, 1–2 days, 3–5 days, 6–8 days and 9 days or more. Child’s T.V. viewing was categorised from the question “on a normal weekday during term time, how many hours does the study child spend watching television, videos or DVDs?” responses coded as; low T.V viewing (0 to <1 h), moderate (≥1 to <3 h) and high (≥3 to 7 h). Parent’s education was used as a proxy of family socio-economic status and was recoded into four categories. The four education categories were i) primary education/lower second level, ii) higher second level education, iii) post second level non-degree and iv) third level education. Measured parent’s BMI categorised as stated previously [25].

### Statistical analyses

Data was analysed using Stata 12 IC (StataCorp LP, USA). Probability weights were applied to account for the complex survey design. Mean differences in DQS between normal weight, overweight and obese children were assessed using an adjusted Wald’s test. The prevalence of normal weight, overweight and obesity was obtained using descriptive statistics with the ‘svy’ prefix. Associations between DQS quintiles and child weight status and other categorical covariates were displayed in contingency tables and differences were tested using the chi^2^ test. A *p*-value < 0.05 was considered statistically significant, tests of hypothesis were two-tailed. Unadjusted and adjusted multinomial logistic regression was used to explore associations between DQS (continuous and in quintiles) and child weight status (normal weight was the reference category). Separate unadjusted and adjusted multinomial logistic regression was also used to explore associations between individual foods (components of the DQS) and child weight status. Tests for trend in the ORs were assessed using the chi-square test for trend. Regression models adjusted for potential confounders in three models; model 1 adjusted for socio-demographic factors (child’s gender and parent’s education), model 2 adjusted for child lifestyle factors; (PA and TV) and model 3 adjusted for parent’s BMI.

### Ethics of human subject participation

the data used for this manuscript was approved by Heath Research Board’s Research Ethics Committee and conforms with the World Medical Association (2000) Declaration of Helsinki: Ethical Principles for Medical Research Involving Human Subjects, with notes of clarification of 2002 and 2004.

## Results

### Demographics

The results that follow include children with valid BMI data only (*N* = 8,136). The following data is not displayed in tables: the DQS ranged from −5 to 25. There was no significant difference between gender and DQS (*p* > 0.436). The mean and standard deviation of the DQS for the whole sample was 9.46 (4.18) and for normal weight, overweight and obese was 9.55 (4.23), 9.46 (4.06) and 8.49 (3.78) respectively. There was a significant difference in the mean DQS for obese children compared to normal weight (*p* < 0.0001) and obese versus overweight children (*p* < 0.0001) but there was no significant differences in mean DQS between normal weight and overweight children. The parent reported DQS was moderately correlated with the child reported DQS (*ρ* = 0.49).

Table 1 presents the prevalence of normal weight, overweight and obesity for the whole sample, in each DQS quintile and by the potential confounders. There was a significant difference between BMI category and gender with more girls categorised as obese compared to boys (Table 1). As the DQS quintile decreased from the highest to the lowest quintile, the percentage of children who were obese increased (4.2 % in the highest quintile and 8.8 % in the lowest quintile, Table 1).

**Table 1:** Prevalence of BMI category for sample and within diet quality, socio-demographic and lifestyle factors, number (percent)

Child BMI	Sample *N* = 8,136		Normal weight 5993 (74.1)	Overweight 1565 (19.3)	Obese 531 (6.6)	
			N (%)			*p*-value
Diet Quality Score	Q1 (Highest Diet Quality)	1,375 (17.0)	1,052 (76.5)	265 (19.3)	58 (4.2)	0.008
	Q2	1,813 (22.4)	1,365 (75.3)	344 (19.0)	104 (5.8)	
	Q3	1,606 (19.9)	1,164 (72.5)	329 (20.5)	113 (7.1)	
	Q4	1,390 (17.2)	1,045 (75.2)	256 (18.5)	89 (6.4)	
	Q5 (Lowest Diet Quality)	1,906 (23.6)	1,368 (71.8)	371 (19.5)	168 (8.8)	
Gender						
	Boys	4,150 (51.3)	3,236 (78.0)	690 (16.6)	224 (5.4)	<0.001
	Girls	3,940 (48.7)	2,758 (70.0)	875 (22.2)	307 (7.8)	
Physical Activity						
	9 days or more	4,426 (54.7)	3,428 (77.4)	800 (18.1)	199 (4.5)	<0.001
	6–8 days	1,568 (19.4)	1,166 (74.4)	300 (19.1)	101 (6.5)	
	3–5 days	1,445 (17.9)	968 (67.0)	339 (23.4)	138 (9.6)	
	1–2 days	462 (5.7)	316 (68.3)	90 (19.5)	57 (12.2)	
	None	187 (2.3)	114 (61.1)	36 (19.5)	37 (19.5)	
Television Viewing						
	0 to <1 h	1,899 (23.5)	1,485 (78.2)	336 (17.7)	79 (4.1)	<0.001
	1 to <3 h	5,324 (65.8)	3,933 (73.9)	1,021 (19.2)	370 (6.9)	
	3 h or more	866 (10.7)	576 (66.5)	207 (23.9)	83 (9.6)	
Parent’s Education						
	Third level	1,378 (17.0)	1,105 (80.1)	230 (16.7)	44 (3.2)	<0.001
	Non-degree	1,304 (16.1)	934 (75.4)	246 (18.8)	75 (5.7)	
	Higher second level	2,987 (36.9)	2,221 (74.5)	578 (19.4)	182 (6.1)	
	<= Lower secondary level	2,426 (30.0)	1,684 (69.4)	511 (21.1)	231 (9.5)	
Parent’s Body Mass Index						
	Normal weight	3,651 (47.8)	3,037 (83.2)	522 (14.3)	92 (2.5)	<0.001
	Overweight	2,479 (32.5)	1,750 (70.6)	533 (21.5)	195 (7.9)	
	Obese	1,506 (19.7)	897 (59.6)	410 (27.2)	200 (13.2)	

Percentages displayed in row %. *p*-values are two sided and significant at <0.05 level

Children with missing BMI values were excluded (*N* = 432, 5.0 %) from all analyses. Numbers may not add up to 8,136 due to survey weighting

Table 2 presents unadjusted odds of obesity associated with diet quality and other covariates. Diet quality is displayed in continuous and categorical form. The unadjusted OR of obesity associated with a one standard deviation increase in DQS was 0.78 (0.70 0.87).

**Table 2:** Independent associations of diet quality, socio-demographic and lifestyle factors on overweight or obesity, number (percent) and odds ratio (95 % confidence intervals)

N (%)		Overweight 1565 (19.3)	Obese 531 (6.6)
		OR (95 % CI)	
Diet Quality Score (DQS continuous)		0.99 (0.98 1.01)	0.94 (0.92 0.97)
Z-DQS^a^		0.98 (0.91 1.05)	0.78 (0.70 0.87)
DQS (quintiles)	Q1 (Highest Diet Quality)	1.00	1.00
	Q2	1.00 (0.80 1.25)	1.40 (0.91 2.15)
	Q3	1.12 (0.89 1.41)	1.78 (1.16 2.73)
	Q4	0.97 (0.77 1.23)	1.55 (1.02 2.36)
	Q5 (Lowest Diet Quality)	1.08 (0.86 1.35)	2.24 (1.50 3.35)
Gender	Boys	1.00	1.00
	Girls	1.49 (1.29 1.72)	1.61 (1.27 2.03)
Parent Education	Third level	1.00	1.00
	Non-degree	1.20 (0.97 1.47)	1.92 (1.22 3.02)
	Higher second level	1.25 (1.03 1.51)	2.08 (1.38 3.13)
	<= Lower secondary level	1.45 (1.17 1.80)	3.47 (2.30 5.21)
Physical Activity	9 days or more	1.00	1.00
	6–8 days	1.10 0.93 1.31)	1.50 (1.12 2.00)
	3–5 days	1.50 (1.25 1.80)	2.46 (1.83 3.31)
	1–2 days	1.22 (0.88 1.70)	3.09 (1.98 4.82)
	None	1.37 (0.87 2.16)	5.50 (3.27 9.27)
Television Viewing	0 to <1 h	1.00	1.00
	1 to <3 h	1.15 (0.96 1.37)	1.77 (1.27 2.47)
	3 h or more	1.59 (1.23 2.05)	2.73 (1.80 4.14)
Parent Body Mass Index category	Normal weight	1.00	1.00
	Overweight	1.77 (1.49 2.10)	3.68 (2.65 5.12)
	Obese	2.66 (2.18 3.23)	7.32 (5.23 10.25)

Unadjusted odds ratios, reference category dependent variable: normal weight

^a^a one standard deviation increase in DQS. All variables listed in the table were analysed in separate models with chid BMI category

### DQS multivariate analyses

Model 1 adjusting for socio-demographic factors showed a significant linear trend in the odds of obesity across DQS quintiles (*p* = 0.007), (Table 3). After adjusting for child’s gender, PA level, TV and parent education, the poorest diet quality (Q5) was associated with an increased odds of obesity by 56 % compared to the highest diet quality (Model 2, Table 3). Further adjustment for parent’s BMI showed a similar though non-significant increase in the odds of obesity in the poorest compared to the highest diet quality (OR 1.32 95 % CI 0.85 2.04). All independent variables were associated with increased odds of obesity (model 3, Table 3). Caution should be taken when interpreting the odds of overweight. The adjusted odds of obesity with a one standard deviation increase in DQS was statistically significant after adjusting for gender, parent education and PA (OR 0.87 95%CI 0.77 0.98) on further inclusion of TV viewing and parent BMI the results were non-significant. Multivariate analyses were also explored using the child answered DQS this score yielded similar results to the parent reported DQS though no significant associations were found in adjusted models (Additional file 2).

**Table 3:** Adjusted association of diet quality, socio-demographic and lifestyle factors on childhood overweight or obesity, odds ratio (95 % confidence intervals)

N (%)		Model 1		Model 2		Model 3	
		Demographic factors		Lifestyle factors		Parental factor	
		Overweight 1565 (19.3)	Obese 531 (6.6)	Overweight	Obese	Overweight	Obese
		OR (95 % CI)					
DQS	Q1 (Highest DQ)	1.00	1.00	1.00	1.00	1.00	1.00
	Q2	0.99 (0.79 1.23)	1.33 (0.86 2.04)	0.98 (0.79 1.23)	1.27 (0.83 1.94)	0.92 (0.72 1.17)	1.10 (0.70 1.72)
	Q3	1.08 (0.85 1.36)	1.57 (1.03 2.41)	1.07 (0.85 1.36)	1.52 (0.99 2.33)	1.04 (0.80 1.34)	1.31 (0.83 2.06)
	Q4	0.95 (0.75 1.20)	1.36 (0.90 2.06)	0.93 (0.74 1.18)	1.23 (0.81 1.88)	0.88 (0.68 1.14)	1.13 (0.72 1.75)
	Q5 (Lowest DQ)	1.01 (0.79 1.27)	1.81 (1.21 2.72)	0.99 (0.78 1.25)	1.56 (1.03 2.37)	0.91 (0.71 1.17)	1.32 (0.85 2.04)
Gender	Boys	1.00	1.00	1.00	1.00	1.00	1.00
	Girls	1.47 (1.28 1.69)	1.57 (1.24 1.97)	1.43 (1.24 1.65)	1.41 (1.11 1.79)	1.44 (1.24 1.67)	1.39 (1.08 1.79)
Parent education							
	Third level	1.00	1.00	1.00	1.00	1.00	1.00
	Non-degree	1.20 (0.97 1.47)	1.88 (1.19 2.96)	1.20 (0.97 1.47)	1.79 (1.13 2.84)	1.15 (0.93 1.43)	2.11 (1.36 3.27)
	Higher second level	1.23 (1.01 1.50)	1.92 (1.27 2.90)	1.22 (1.00 1.49)	1.78 (1.17 2.70)	1.20 (0.97 1.47)	2.15 (1.48 3.14)
	<= Lower secondary level	1.42 (1.14 1.78)	3.01 (1.98 4.58)	1.41 (1.13 1.77)	2.70 (1.78 4.09)	1.26 (0.99 1.59)	2.78 (1.89 4.10)
Physical Activity	9 days or more			1.00	1.00	1.00	1.00
	6–8 days			1.06 (0.89 1.26)	1.45 (1.08 1.95)	1.06 (0.89 1.28)	1.47 (1.07 2.02)
	3–5 days			1.40 (1.16 1.68)	2.23 (1.63 3.04)	1.38 (1.14 1.67)	2.25 (1.64 3.09)
	1–2 days			1.08 (0.77 1.51)	2.51 (1.59 3.96)	1.09 (0.76 1.55)	2.28 (1.38 3.76)
	None			1.18 (0.74 1.87)	4.34 (2.54 7.42)	1.20 (0.75 1.92)	4.57 (2.61 8.00)
Television viewing	Low			1.00	1.00	1.00	1.00
	Moderate			1.11 (0.93 1.33)	1.51 (1.09 2.11)	1.05 (0.87 1.26)	1.38 (0.97 1.93)
	High			1.47 (1.13 1.92)	1.81 (1.16 2.82)	1.38 (1.05 1.83)	1.71 (1.08 2.71)
Parent Body Mass Index category	Normal					1.00	1.00
	Overweight					1.76 (1.48 2.08)	3.51 (2.52 4.90)
	Obese					2.60 (2.12 3.16)	6.48 (4.59 9.14)

Reference categories: child BMI (dependant variable): normal weight, Reference category (independent variables): DQS Q1 (Highest diet quality), gender = Male, parent’s education = third level, PA = 9 days or more PA, T.V. viewing = Low (0 to <1 h), parent’s BMI = normal weight

Model 1 adjusted for child gender and parent’s education, Model 2 adjusted Model 1, child’s PA level, and T.V. viewing, Model 3 adjusted for Models 1 and 2 and parent’s BMI category

### Analyses of individual foods

The association between the individual food components of the DQS and overweight and obesity are displayed in additional file 3. Individual food components such as; cooked and raw vegetables/salad, potatoes /rice/ pasta, cereals, full cream milk/ milk products, crisps/savoury snacks, biscuits/ doughnuts/ cake/ pie/ chocolate, were all negatively associated with overweight and obesity in fully adjusted models (Additional file 3). Results of the child reported components of the DQS were also analysed and were similar to the parent reported components (Additional file 4).

## Discussion

The present research aimed to investigate the association between diet quality and childhood overweight or obesity, constructed from a short dietary assessment tool. The main finding was that a simple DQS constructed from a 20 item, parent reported, FFQ of foods eaten over the past 24-h was associated with childhood obesity. This easy to administer, short FFQ, with a simple application to a DQS, may be a useful tool in other large-scale epidemiological studies to identify children with poor dietary patterns. The findings also suggest that diet quality, as opposed to individual foods or food groups, may act as a useful explanatory variable in the complex myriad of factors associated with the development of obesity. Many foods or nutrients may be involved in the promotion or protection against obesity [32]. When only one food or nutrient is studied in relation to an outcome such as obesity the findings are often inconsistent across studies [32]. Individual foods or nutrients may be captured with less precision/reliability especially when investigating obesity as there is a trend to under-report certain foods by overweight participants [32-34]. ‘No single nutrient has been unequivocally associated with the development of obesity’ [32] and hence DQS are being used more frequently as they reflect overall dietary patterns better than single nutrients or food groups [35] and they take into account that the diet as a whole may be promoting obesity rather than one food/nutrient.

There are several other advantages to assessing the diet-disease relationship using DQS such as; ‘the effect of a single nutrient may be too small to detect, but the cumulative effects of multiple nutrients included in a dietary pattern may be sufficiently large to be detectable’ [16, 15]. The approach to examining diet-disease relationships using DQS is particularly useful where multiple dietary components are relevant for a particular disease, as it accounts for interactions and correlations between foods and food groups. The approach may be particularly useful for Coronary Heart Disease (CHD), hypertension, diabetes mellitus and obesity where multiple dietary components are established risk factors [15]. Our DQS demonstrated sufficient power to detect differences in diet quality between normal weight and obese children, differences in diet quality between normal weight and overweight children were less pronounced. The results for overweight are not as pronounced for diet quality but also the other covariates. It is difficult to interpret the comparison of the overweight versus normal weight children as an increased risk of obesity causes a reduction in risk of overweight. It is likely that as a risk factor increases the risk of obesity this has made the children less likely to be overweight as they are more likely to be obese. Whilst our DQS may not provide a reliable estimate of individual diet, it does provide estimates of mean differences in diet quality between groups of normal weight and obese children. Though individuals’ diets vary day to day, we would expect that the mean for the group would be stable. Moreover, variation in time between groups would be uniform. We are of the opinion that our DQS shows construct validity, as stated previously no formal definition of diet quality exists, however DQS are used in the literature as measures of diet quality.

Our findings are similar to those from other studies on diet quality and childhood obesity that used more detailed measures to assess diet such as a 132 item FFQ [36] 4 day food diary [37], and a 154 item FFQ [38]. Feskanich et al. [36], found that a higher DQS measured using a modified version of the Health Eating Index was associated with a reduced BMI in children [36]. In the UK, Jennings et al. [37], found that the Diet Quality Index (DQI) and the Health Diet Indicator (HDI) were both associated with lower waist circumference and lower body fat in a sample of children similar to those included in the current study. The DQI was also associated with lower BMI after controlling for confounding factors such as PA and overall energy density [37]. The Mediterranean diet KIDMED index was also negatively associated with obesity status though physical activity was suggested as a mediator of the association [38]. The KIDMED study differs from the current study in a number of important respects. The assessment of PA was more rigorous in the KIDMED study but the investigators relied on parent reported child height and weight. This group investigated BMI as a binary outcome, categorising overweight and obesity together. In the current study where we examined BMI as a continuous predictor and as a 3 level categorical variable, there is more variability in diet quality between obese and normal weight children than overweight and normal weight children. This suggests that it is best (sample size permitting) not to combine the overweight and obese categories, as associations with obesity may be missed. Similar to our study, Lazarou et al. [38] found that maternal obesity was a strong predictor of childhood obesity. A possible explanation is that parent BMI may be regarded as a proxy measure of diet quality in childhood, reflecting shared eating environments.

A review of short tools to assess children aged 2–5 years’ dietary intake, found that short dietary tools to screen for obesogenic dietary behaviours could be judged useful [21]. This review also stated that dietary assessment tools from which DQS are developed should include the five major food groups as well as foods deemed unhealthy in order to assess diet quality. The measure we used in the current study, although short, did include the five major food groups, i.e. fruit and vegetables, grains, meat and alternatives as well as measures on sugar sweetened beverages and snacks, and foods high in saturated fat, see Additional file 1.

In order to address our secondary aim we investigated the individual food components of the DQS to identify if any particular foods were driving the DQS. Some of the foods were negatively associated with child obesity as expected. However, foods such as crisps/ savoury snacks and biscuits/ doughnuts/ cakes/ pie/ chocolate were associated with lower levels of obesity, which is counter-intuitive. It may be possible that there is more variability between obese and non-obese children in terms of consumption of the healthy DQS food components. Alternatively, the unexpected findings in relation to some specific food items may reflect reporting bias. Furthermore a lack of variability between normal weight and obese children may be down to the dietary assessment method for example the tool did not define the portion size or whether ‘more than once’ was twice, three or many more times. Finally, it may be that both normal weight and obese children consume a high level of unhealthy snack foods.

The strengths of our research include that the DQS was developed on a large nationally representative sample of children and their families (*n* = 8568). All analyses took into account the complex survey design by applying survey weights. We controlled for some important confounders, though residual confounding is possible. The dietary assessment tool was completed with a low level of missing data for individual items, suggesting that this tool is easily administered with low respondent burden. The underlying parent reported FFQ was similar to the child reported FFQ and this may suggest that parents can provide useful data on their child’s diet. Parents as proxy reporters of their child’s diet has been suggested as guideline to adhere to for children less than 10 years old [39].

Our research also had limitations. Causal inference cannot be implied, given the cross-sectional nature of the study. There is a possibility that a child’s weight status may have introduced response bias from parents. The underlying short FFQ was not tested for validity or reliability. However there are limitations in assessing diets in any population regardless of the assessment technique as detailed by Magarey et al., 2011 [39]. It should also be noted that there is no gold standard method to measure diet or diet quality. All studies of human food intake, under natural conditions reveal a substantial degree of day to day variability [40] and misreporting [34]. Similarly techniques to assess reliability or validity of various short dietary assessment tools also have limitations as described by Bell et al. (2013), in particular the over-reliance on correlations coefficients [21]. An Australian short dietary assessment tool that tested validity and reliability was conducted [41] however not all core food groups were assessed. Future research studies that validate DQS’ against more detailed dietary assessments, taking day to day variability, energy misreporting and overall energy intake into account, together with validating it against biological biomarkers, may be the best approximation of diet quality [42]. To our knowledge no such validated DQS, for use in children, focusing on health outcomes, developed from short dietary assessments, exist.

Simple tools to assess diet quality and identify children who follow unhealthy dietary patterns are important for developing appropriate childhood obesity interventions [43]. As diet patterns [44] and obesity [4] can track into adulthood, promoting healthy eating patterns in childhood may be a useful preventative strategy. Not all studies can afford to assess children’s diet using dietary recalls, food diaries or long FFQ’s. The short FFQ used in this research was easy to understand and cost effective in terms of expertise needed by researchers to both collect and enter the data. This study adds to the emerging area on whole diet analysis and its association with childhood obesity. Research in this area to date is largely based on in-depth scores that, prior to construction, require a more detailed picture of the child’s diet based on methods such as food diaries [37], 24 h dietary recall [45] and 132 item FFQ [36]. These methods although preferred are not always practical to use in large survey designs. Some research using shorter tools are emerging [21]. However, these instruments, like the DQS used in the current study, need more comprehensive reliability and validity testing.

## Conclusion

In conclusion, a simple diet quality score was associated with obesity but not overweight in a large representative sample of 9-year old children. Short dietary assessment instruments, such as the DQS used in this study, may provide an acceptable and effective approach to the study of diet disease associations in large studies where there is a need to minimise respondent burden or when resources are scarce. Parent’s BMI is an important factor in childhood overweight and obesity and it is clear that the influence of parental BMI on children’s diet warrants further investigation. Although individual foods items were associated with overweight and obesity in children the findings were inconsistent.

## Additional files

Additional file 1:
**Dietary Assessment tool Growing up in Ireland Mother or Lone Father questionnaire and the child main questionnaire.**
Additional file 2:
**Prevalence odds ratios for overweight and obesity with frequency of consumption of individual foods component of parent reported DQS.pdf.**
Additional file 3:
**Prevalence odds ratios for overweight and obesity with frequency of consumption of individual food components of child reported DQS.pdf.**
Additional file 4:
**Prevalence odds ratios for overweight and obesity with frequency of consumption of individual food components of child reported DQS.**

## Abbreviations

BMI: Body mass index
DQS: Dietary quality score
GUI: Growing up in Ireland
IOTF: International obesity taskforce
PA: Physical activity
ROI: Republic of Ireland
TV: Television
AHA: Amherst health and activity study
