# Supplementary material for: The use of a dietary quality score as a predictor of childhood overweight and obesity
Source: BMC Public Health. 2015 Jun 24;15:581. doi: 10.1186/s12889-015-1907-y (PMC4477494; doi:10.1186/s12889-015-1907-y)
Supplement: Additional file 1: — Dietary Assessment tool Growing up in Ireland Mother or Lone Father questionnaire and the child main questionnaire. [file 12889_2015_1907_MOESM1_ESM.pdf]

Additional File 1 (a): Dietary Assessment tools Growing up in Ireland Mother or Lone Father questionnaire

D. CHILD'S DIET AND EXERCISE

D1. *[Show Card D1]* **Looking at Card D1, in the last 24 hours has the Study Child had the following foods and drinks once, more than once, or not at all?**

	Once	More than Once	Not At All	Don't know
A. Fresh fruit	☐ 1	☐ 2	☐ 3	☐ 4
B. Fruit juice	☐ 1	☐ 2	☐ 3	☐ 4
C. Meat / Chicken / Fish	☐ 1	☐ 2	☐ 3	☐ 4
D. Eggs	☐ 1	☐ 2	☐ 3	☐ 4
E. Cooked vegetables	☐ 1	☐ 2	☐ 3	☐ 4
F. Raw vegetables or salad	☐ 1	☐ 2	☐ 3	☐ 4
G. Meat pie, hamburger, hot dog, sausage or sausage roll	☐ 1	☐ 2	☐ 3	☐ 4
H. Hot chips or French fries	☐ 1	☐ 2	☐ 3	☐ 4
I. Crisps or savoury snacks	☐ 1	☐ 2	☐ 3	☐ 4
J. Bread	☐ 1	☐ 2	☐ 3	☐ 4
K. Potatoes/ Pasta/ Rice	☐ 1	☐ 2	☐ 3	☐ 4
L. Cereals	☐ 1	☐ 2	☐ 3	☐ 4
M. Biscuits, doughnuts, cake, pie or chocolate	☐ 1	☐ 2	☐ 3	☐ 4
N. Cheese/yoghurt/ fromage frais	☐ 1	☐ 2	☐ 3	☐ 4
O. Low fat Cheese/ low fat yoghurt	☐ 1	☐ 2	☐ 3	☐ 4
P. Water (tap water / still water/ sparkling water)	☐ 1	☐ 2	☐ 3	☐ 4
Q. Soft drinks / minerals / cordial / squash (not diet)	☐ 1	☐ 2	☐ 3	☐ 4
R. Soft drinks / minerals / cordial / squash (diet)	☐ 1	☐ 2	☐ 3	☐ 4
S. Full cream milk or full cream milk products	☐ 1	☐ 2	☐ 3	☐ 4
T. Skimmed milk or skimmed milk products	☐ 1	☐ 2	☐ 3	☐ 4

Additional File 1 (b): Dietary Assessment tool Growing up in Ireland child main questionnaire

6. We would like you to think back to what you ate yesterday. Did you eat the following?

	No	One Serving	More than one serving
a. Fresh fruit	☐ 1	☐ 2	☐ 3
b. Cooked vegetables	☐ 1	☐ 2	☐ 3
c. Meat pie, hamburger, hot dog, sausage or sausage roll (any of these)	☐ 1	☐ 2	☐ 3
d. Chips or French fries	☐ 1	☐ 2	☐ 3
e. Crisps or savoury snacks	☐ 1	☐ 2	☐ 3
f. Biscuits, doughnuts, cake, pie or chocolate (any of these)	☐ 1	☐ 2	☐ 3
g. Milk	☐ 1	☐ 2	☐ 3
h. Cheese or yoghurt	☐ 1	☐ 2	☐ 3
i. Fizzy drinks or diet drinks	☐ 1	☐ 2	☐ 3
j. Bread, Pasta, Rice, Cereal (any of these)	☐ 1	☐ 2	☐ 3
