# Supplementary material for: The use of a dietary quality score as a predictor of childhood overweight and obesity
Source: BMC Public Health. 2015 Jun 24;15:581. doi: 10.1186/s12889-015-1907-y (PMC4477494; doi:10.1186/s12889-015-1907-y)
Supplement: Additional file 2: — Prevalence odds ratios for overweight and obesity with frequency of consumption of individual foods component of parent reported DQS.pdf. [file 12889_2015_1907_MOESM2_ESM.pdf]

Additional file 2: Prevalence odds ratios for overweight and obesity with Child DQS‡ and potential confounders

‡ derived from child's answers to 10-item FFQ of foods eaten yesterday in child main questionnaire. Model 1 unadjusted model, Model 2Adjusted for gender and parent's education,

		N (%)							
		**Model 1 Unadjusted**		**Model 2 Demographics factors**		**Model 3 Lifestyle factors**		**Model 4 Parent factor**	
	**N (%)**	**Overweight** 1,565 (19.3)	**Obese** 531 (6.6)	**Overweight**	**Obese**	**Overweight**	**Obese**	**Overweight**	**Obese**
		**OR (95 % CI)**							
**DQS**	**Q1 (Highest DQ)**	1.00	1.00	1.00	1.00	1.00	1.00	1.00	1.00
	**Q2**	1.09 (0.85 1.39)	1.00 (0.65 1.53)	1.07 (0.83 1.37)	0.95 (0.62 1.46)	1.05 (0.82 1.35)	0.92 (0.60 1.41)	1.08 (0.84 1.40)	0.99 (0.62 1.60)
	**Q3**	1.03 (0.81 1.30)	1.09 (0.71 1.66)	0.99 (0.78 1.26)	0.99 (0.64 1.53)	0.99 (0.77 1.26)	0.96 (0.61 1.49)	1.00 (0.78 1.29)	1.06 (0.67 1.68)
	**Q4**	1.02 (0.84 1.24)	1.50 (1.05 2.12)	0.98 (0.81 1.19)	1.32 (0.93 1.88)	0.96 (0.79 1.16)	1.24 (0.87 1.77)	0.93 (0.76 1.13)	1.24 (0.85 1.83)
	**Q5 (Lowest DQ)**	0.93 (0.75 1.14)	1.27 (0.87 1.84)	0.86 0.69 1.07	1.03 (0.70 1.51)	0.82 (0.66 1.02)	0.91 (0.62 1.36)	0.79 (0.63 0.99)	0.88 (0.58 1.33)
**Gender**									
	**Boys**			1.00	1.00	1.00	1.00	1.00	1.00
	**Girls**			1.47 (1.28 1.70)	1.56 (1.24 1.97)	1.44 (1.25 1.67)	1.41 (1.11 1.78)	1.44 (1.24 1.67)	1.39 (1.09 1.79)
**Parent education**									
	**Third level**			1.00	1.00	1.00	1.00	1.00	1.00
	**Non-degree**			1.21 (0.98 1.48)	1.89 (1.21 2.97)	1.18 (0.96 1.45)	1.80 (1.14 2.83)	1.16 (0.93 1.44)	2.10 (1.36 3.24)
	**Higher second level**			1.26 (1.03 1.53)	2.01 (1.33 3.03)	1.21 (1.00 1.47)	1.83 (1.21 2.78)	1.22 (0.99 1.49)	2.21 (1.52 3.22)
	**<= Lower secondary level**			1.47 (1.18 1.83)	3.31 (2.20 4.99)	1.40 (1.12 1.75)	2.91 (1.93 4.37)	1.30 (1.03 1.63)	2.97 (2.03 4.36)
**PA**									
	**9 days or more**					1.00	1.00	1.00	1.00
	**6-8 days**					1.05 (0.88 1.25)	1.45 (1.08 1.95)	1.05 (0.88 1.27)	1.47 (1.06 2.02)
	**3-5days**					1.39 (1.16 1.67)	2.26 (1.66 3.08)	1.38 (1.14 1.66)	2.29 (1.67 3.14)
	**1-2 days**					1.08 (0.77 1.52)	2.56 (1.63 4.02)	1.09 (0.76 1.55)	2.34 (1.42 3.84)
	**None**					1.19 (0.75 1.89)	4.37 (2.56 7.48)	1.22 (0.76 1.95)	4.60 (2.62 8.09)
**TV**	**Low**					1.00	1.00	1.00	1.00
	**Moderate**					1.12 (0.93 1.34)	1.56 (1.11 2.18)	1.05 (0.87 1.27)	1.38 (0.98 1.96)
	**High**					1.51 (1.16 1.97)	1.90 (1.21 2.98)	1.42 (1.08 1.88)	1.78 (1.13 2.82)
**Parent BMI**	**Normal**							1.00	1.00
	**Overweight**							1.77 (1.49 2.10)	3.53 (2.54 4.91)
	**Obese**							2.62 (2.15 3.20)	6.64 (4.70 9.37)

Model 3 Adjusted model 2 and physical activity, and child's T.V. viewing time Model 4 adjusted for models 2 and 3 and parent's BMI
