# Supplementary material for: The use of a dietary quality score as a predictor of childhood overweight and obesity
Source: BMC Public Health. 2015 Jun 24;15:581. doi: 10.1186/s12889-015-1907-y (PMC4477494; doi:10.1186/s12889-015-1907-y)
Supplement: Additional file 3: — Prevalence odds ratios for overweight and obesity with frequency of consumption of individual food components of child reported DQS.pdf. [file 12889_2015_1907_MOESM3_ESM.pdf]

Additional file 3: Prevalence odds ratios for overweight and obesity with frequency of consumption of individual foods component of parent reported DQS

		Model 1 †		Model 2 ††	
OR (95 % CI)		Overweight	Obese	Overweight	Obese
**Water**	**Eaten once**<br>**Eaten > once**	0.89 (0.68 1.15)<br>1.15 (0.93 1.42)	1.25 (0.83 1.89)<br>1.14 (0.81 1.61)	0.90 (0.69 1.19)<br>1.22 (0.97 1.53)	1.25 (0.81 1.92)<br>1.32 (0.92 1.90)
**Fresh fruit**	**Eaten once**<br>**Eaten > once**	1.16 (0.97 1.39)<br>1.00 (0.84 1.20)	1.22 (0.90 1.64)<br>0.85 (0.62 1.16)	1.17 (0.96 1.42)<br>1.02 (0.84 1.24)	1.35 (0.97 1.87)<br>1.13 (0.80 1.59)
**Fruit juice**	**Eaten once**<br>**Eaten > once**	1.06 (0.87 1.29)<br>1.09 (0.89 1.33)	1.14 (0.85 1.52)<br>0.90 (0.66 1.23)	1.05 (0.86 1.29)<br>1.10 (0.90 1.35)	1.12 (0.81 1.54)<br>0.98 (0.70 1.37)
**Cooked veg**	**Eaten once**<br>**Eaten > once**	0.98 (0.83 1.16)<br>0.85 (0.69 1.05)	0.86 (0.66 1.13)<br>0.60 (0.43 0.83)	1.01 (0.84 1.20)<br>0.90 (0.72 1.12)	0.98 (0.74 1.30)<br>0.79 (0.56 1.13)
**Raw veg**	**Eaten once**<br>**Eaten > once**	1.09 (0.91 1.32)<br>0.92 (0.68 1.25)	0.60 (0.44 0.83)<br>0.55 (0.34 0.91)	1.07 (0.88 1.31)<br>0.87 (0.62 1.23)	0.66 (0.47 0.93)<br>0.54 (0.32 0.93)
**Meat/ chicken/ fish**	**Eaten once**<br>**Eaten > once**	1.09 (0.77 1.55)<br>0.98 (0.69 1.40)	0.87 (0.54 1.39)<br>0.64 (0.40 1.05)	1.11 (0.77 1.58)<br>1.02 (0.71 1.48)	1.02 (0.60 1.71)<br>0.84 (0.49 1.44)
**Eggs**	**Eaten once**<br>**Eaten > once**	1.02 (0.85 1.22)<br>0.76 (0.51 1.12)	1.02 (0.75 1.39)<br>0.83 (0.46 1.50)	0.97 (0.80 1.17)<br>0.74 (0.48 1.13)	1.01 (0.72 1.39)<br>0.87 (0.46 1.65)
**Cereals**	**Eaten once**<br>**Eaten > once**	0.82 (0.66 1.01)<br>0.71 (0.56 0.91)	0.67 (0.49 0.92)<br>0.45 (0.31 0.65)	0.85 (0.68 1.07)<br>0.78 (0.61 1.01)	0.74 (0.53 1.04)<br>0.50 (0.34 0.75)
**Potato/ pasta/ rice**	**Eaten once**<br>**Eaten > once**	0.90 (0.73 1.10)<br>0.88 (0.69 1.11)	0.81 (0.58 1.12)<br>0.56 (0.38 0.83)	0.89 (0.72 1.10)<br>0.93 (0.73 1.19)	0.94 (0.66 1.34)<br>0.65 (0.42 1.00)
**Bread**	**Eaten once**<br>**Eaten > once**	1.00 (0.76 1.33)<br>0.85 (0.64 1.14)	1.13 (0.72 1.78)<br>0.84 (0.53 1.35)	1.10 (0.82 1.46)<br>0.97 (0.73 1.30)	1.36 (0.83 2.22)<br>1.06 (0.64 1.75)
**Full cream milk**	**Eaten once**<br>**Eaten > once**	0.70 (0.58 0.85)<br>0.66 (0.56 0.78)	0.61 (0.46 0.81)<br>0.44 (0.34 0.58)	0.68 (0.56 0.83)<br>0.70 (0.59 0.84)	0.65 (0.48 0.88)<br>0.53 (0.39 0.71)
**Skimmed milk**	**Eaten once**<br>**Eaten > once**	1.79 (1.36 2.34)<br>1.76 (1.40 2.21)	2.09 (1.44 3.05)<br>1.74 (1.20 2.52)	1.75 (1.31 2.33)<br>1.86 (1.46 2.36)	1.90 (1.26 2.87)<br>1.89 (1.27 2.82)
**Cheese/ yoghurt**	**Eaten once**<br>**Eaten > once**	1.05 (0.89 1.23)<br>0.96 (0.80 1.16)	0.79 (0.60 1.05)<br>0.79 (0.58 1.10)	1.07 (0.90 1.27)<br>1.00 (0.81 1.22)	0.84 (0.62 1.14)<br>0.85 (0.60 1.20)
**Low fat cheese/ yoghurt**	**Eaten once**<br>**Eaten > once**	1.77 (1.36 2.29)<br>1.66 (1.07 2.58)	2.22 (1.56 3.14)<br>2.14 (1.19 3.82)	1.66 (1.25 2.21)<br>1.75 (1.08 2.83)	1.84 (1.23 2.73)<br>2.23 (1.18 4.20)
**Meat pie sausage**	**Eaten once**<br>**Eaten > once**	0.97 (0.83 1.15)<br>0.96 (0.66 1.38)	1.24 (0.96 1.59)<br>1.50 (0.94 2.39)	0.97 (0.82 1.15)<br>0.95 (0.64 1.41)	1.06 (0.81 1.39)<br>1.29 (0.77 2.17)
**Hot chips**	**Eaten once**<br>**Eaten > once**	1.12 (0.97 1.29)<br>1.37 (0.87 2.16)	1.17 (0.92 1.50)<br>1.24 (0.66 2.31)	0.97 (0.83 1.13)<br>1.18 (0.69 2.01)	0.88 (0.68 1.15)<br>0.75 (0.36 1.55)
**Crisps/ savoury snacks**	**Eaten once**<br>**Eaten > once**	0.97 (0.83 1.13)<br>0.92 (0.74 1.15)	1.00 (0.77 1.30)<br>0.64 (0.43 0.94)	0.92 (0.78 1.08)<br>0.84 (0.66 1.07)	0.97 (0.74 1.28)<br>0.50 (0.33 0.75)
**Biscuits/ chocolate**	**Eaten once**<br>**Eaten > once**	0.75 (0.63 0.90)<br>0.69 (0.57 0.84)	0.65 (0.50 0.85)<br>0.62 (0.46 0.84)	0.82 (0.68 0.98)<br>0.74 (0.61 0.91)	0.69 (0.51 0.92)<br>0.67 (0.48 0.93)
**Regular soft drinks**	**Eaten once**<br>**Eaten > once**	1.16 (0.97 1.37)<br>1.00 (0.84 1.19)	0.99 (0.76 1.30)<br>1.02 (0.76 1.38)	1.08 (0.91 1.30)<br>0.87 (0.72 1.05)	0.83 (0.62 1.10)<br>0.79 (0.57 1.10)
**Diet soft drinks**	**Eaten once**<br>**Eaten > once**	1.29 (1.05 1.58)<br>1.39 (1.09 1.77)	2.01 (1.48 2.71)<br>2.63 (1.85 3.73)	1.20 (0.96 1.50)<br>1.37 (1.06 1.78)	1.69 (1.21 2.35)<br>2.12 (1.47 3.05)

Reference category for each food component was not eaten at all over the past 24 hours. †Unadjusted regression †† Adjusted for gender, parent's education, child's PA, child's T.V. viewing, parent's BMI. All individual food components were analysed in separate models with the outcome and confounders.
