# Supplementary material for: The use of a dietary quality score as a predictor of childhood overweight and obesity
Source: BMC Public Health. 2015 Jun 24;15:581. doi: 10.1186/s12889-015-1907-y (PMC4477494; doi:10.1186/s12889-015-1907-y)
Supplement: Additional file 4: — Prevalence odds ratios for overweight and obesity with frequency of consumption of individual food components of child reported DQS. [file 12889_2015_1907_MOESM4_ESM.pdf]

Additional file 4: Prevalence odds ratios for overweight and obesity with frequency of consumption of individual food components of child reported DQS

		Model 1 †		Model 2 ††	
OR (95 % CI)		Overweight	Obese	Overweight	Obese
**Fresh fruit**	**Eaten once**<br>**Eaten > once**	1.01 (0.85 1.19)<br>0.91 (0.74 1.11)	1.05 (0.81 1.36)<br>0.85 (0.61 1.17)	1.03 (0.87 1.23)<br>0.95 (0.77 1.17)	1.09 (0.82 1.44)<br>0.95 (0.68 1.34)
**Cooked veg**	**Eaten once**<br>**Eaten > once**	0.96 (0.83 1.11)<br>0.86 (0.70 1.07)	0.70 (0.55 0.90)<br>0.60 (0.56 1.13)	1.01 (0.87 1.18)<br>0.92 (0.74 1.15)	0.79 (0.61 1.03)<br>0.99 (0.68 1.45)
**Bread, pasta, rice or cereal**	**Eaten once**<br>**Eaten > once**	1.00 (0.77 1.31)<br>1.07 (0.81 1.41)	0.94 (0.64 1.40)<br>0.60 (0.40 0.91)	1.03 (0.78 1.36)<br>1.15 (0.86 1.53)	0.94 (0.61 1.45)<br>0.65 (0.41 1.03)
**Milk**	**Eaten once**<br>**Eaten > once**	1.00 (0.81 1.23)<br>0.95 (0.77 1.16)	0.83 (0.63 1.11)<br>0.59 (0.42 0.82)	1.11 (0.89 1.38)<br>1.10 (0.88 1.37)	0.91 (0.67 1.23)<br>0.67 (0.47 0.95)
**Cheese/ yoghurt**	**Eaten once**<br>**Eaten > once**	1.07 (0.92 1.25)<br>1.13 (0.91 1.41)	1.22 (0.95 1.57)<br>1.14 (0.76 1.70)	1.15 (0.97 1.35)<br>1.18 (0.94 1.49)	1.33 (1.03 1.73)<br>1.27 (0.83 1.95)
**Meat pie sausage**	**Eaten once**<br>**Eaten > once**	0.98 (0.84 1.15)<br>1.17 (0.81 1.67)	1.14 (0.89 1.45)<br>0.65 (0.29 1.46)	0.96 (0.81 1.13)<br>1.16 (0.79 1.70)	0.96 (0.74 1.26)<br>0.66 (0.29 1.46)
**Chips or French fries**	**Eaten once**<br>**Eaten > once**	0.93 (0.80 1.09)<br>0.92 (0.57 1.48)	1.42 (1.12 1.80)<br>0.50 (0.20 1.25)	0.87 (0.74 1.03)<br>0.91 (0.55 1.52)	1.20 (0.93 1.54)<br>0.47 (0.19 1.17)
**Crisps/ savoury snacks**	**Eaten once**<br>**Eaten > once**	0.88 (0.76 1.02)<br>0.78 (0.59 1.01)	1.06 (0.83 1.35)<br>0.64 (0.40 1.03)	0.86 (0.73 1.00)<br>0.72 (0.55 0.95)	1.04 (0.80 1.34)<br>0.54 (0.33 0.89)
**Biscuits/ chocolate**	**Eaten once**<br>**Eaten > once**	0.87 (0.74 1.02)<br>0.72 (0.57 0.90)	0.62 (0.48 0.81)<br>0.55 (0.39 0.80)	0.87 (0.74 1.04)<br>0.71 (0.56 0.90)	0.62 (0.47 0.83)<br>0.52 (0.35 0.78)
**Fizzy drinks**	**Eaten once**<br>**Eaten > once**	0.97 (0.83 1.13)<br>0.94 (0.72 1.19)	1.40 (1.09 1.80)<br>1.24 (0.83 1.87)	0.87 (0.74 1.03)<br>0.86 (0.67 1.10)	1.08 (0.83 1.41)<br>0.91 (0.59 1.42)

Reference category: not eaten over the past 24 hours. †Unadjusted regression †† Adjusted for child's gender, PA level and T.V. viewing and the parent's education and BMI.
